# Modeling Determinants of Health Expenditures in Malaysia: Evidence from Time Series Analysis

**DOI:** 10.3389/fphar.2016.00069

**Published:** 2016-03-30

**Authors:** Habib N. Khan, Radzuan B. Razali, Afza B. Shafie

**Affiliations:** Fundamental and Applied Sciences Department, Applied Statistics/Econometrics, University Tekonologi PetronasTronoh, Malaysia

**Keywords:** health care expenditure, dynamic relationship, diagnostic tests, model specification, auto-regressive distributed lag model, driving forces, granger causality

## Introduction

The purpose of this paper is to model the determinants of health care expenditures (HCE) and investigate the short-run, long-run equilibrium dynamic causal relationship between health care and income per capita within the time series framework from 1981 to 2014 in Malaysia. For appropriate model specification and forecasting accuracy, different econometric diagnostic tests were applied. Ordinary least square (OLS) method was used to estimate the long run parameters. Long run co-integration was investigated by Auto-Regressive Distributed *Lag* Model (ARDL) Bound approach, whereas, for causality analysis the Engle-Granger method was used. Income, population structure, and population growth was identified as the significant contributing factors to explain variations in HCE. The estimated income elasticity for HCE was found 0.99 < 1 showing health care was a necessity. The results confirmed a feed-back hypothesis between health expenditure and income per capita.

Money spending and health care expenditure relationship has long been established (Getzen, 2014). Better health has been identified as an important factor to raise economic growth and increased productivity. A healthy population of any country is of important importance and has positive connections to economic growth (Sachs, 2002; Khan et al., 2015). However, rapidly growing HCE is a matter of grave concern for policy and decision makers across countries in the world. The fast growth rate of health care spending exerts pressure on various sectors of the economy, which might slow down the economic growth sustainability (Jakovljevic and Milovanovic, 2015; Jakovljevic, 2016) create poverty trap, as more out-of-pocket health expenditure hugely affects household income (Khan et al., 2015).

### Health care expenditure and the Malaysia case

Malaysia with a total land area of 329,758 square kilometers is one of the leading and fast growing high middle-income economies in the Southeast Asian countries. The total population of the country is approximately 29,717 million which is distributed within 14 states, with a per capita gross national income of US $22 (international PPP); and life expectancy rate ranging from 72 to 76 years at birth of male and female respectively. It spends US$ 938 billion total on health with a growth rate of more than 4.49% on HCE (WHO, 2013). Malaysia, a rapidly fast growing developing economy in the Southeast Asian countries, spent 2.94, and 4.49% of GDP on its total health expenditure, in 1997 and 2012, respectively. The overall per capita spending over the same period was US $223 and US$463, respectively. In 2012, the sector-wise share of health care financing expenditure was: Ministry of health 44%; out-of-pocket 37%; private insurance 7%; other federal agencies 4% (MOH, 2014). The health expenditure growth rate of 4.49%, when compared to the annual GDP growth rate of 6%, shows the persistent rise in growth of health expenditure which might cause slowing down growth process of economy to a snail's pace. This might exert burden on country's GDP in the form of deficit budget, provision of health care services, and patients out of pocket finances. Thus, it is needed to model and forecast determinants of health care expenditure and future trends in the health care spending, in order to devise appropriate policies to control the rapidly growing HCE growth, equitable health care services provision, and affordable treatments to the people of Malaysia.

This paper aims at, modeling the determinants of health care expenditure (HCE) and the effects of contributing factors of increased health care spending on economic growth by using annual data ranging from 1981 to 2014 in Malaysia.

### Motivation of the study

Based on the current literature survey, this study is first of its kind which attempts to model and investigate factors influencing HCE over an extended time period in Malaysia economy using, ordinary least square (OLS), Autoregressive Distributed Lag Model (ARDL) using annual time series data ranging from 1981 to 2014. We investigate time series properties such as unit root and co-integration between health care and income per capita. Besides, the causality is also examined through Engle Granger (1969) test to find out the direction of causation and for policy implications. The remainder paper is structured as follows:

Section 2 is devoted to an overview of the existing literature on the topic. In section 3, data and sources of data; variables and their measurements are discussed. Moreover, model specification and methodology is also included in this section. Section 4 discusses estimated results and section 5 concludes with some policy implications and suggestions.

## Literature review

### Introduction

Rapid population growth has raised serious concerns about the improvements in health status of the general public, health care systems‘ financial sustainability, both in developed and developing countries as well. Over the last couple of decades, it has been a point of debate, both for applied econometricians and health economists to analyze HCE and its determinants. In addition to that, it has also been very tempting to growth economists and to theoretical and applied econometricians to study economic growth and factors affecting economic growth. The applied econometricians and economists made attempts to model and analyze the relationship between HCE, determinants of HCE, and GDP per capita, including their factors by modeling and analyzing the association between HCE and other non-economic, social and demographic variables at individual country levels and in a panel of countries. Further, accumulated review of the literature on the subject is overviewed in the sections discussed below.

### Major factors of health care expenditure

#### Income as a driver of health care expenditure

Numerous studies investigated the HCE and income relationship in a cross-sectional framework with principal findings (1) Income as a potential factor responsible for explaining variations in level and growth of HCE across countries; (2) health expenditure a luxury good with an income elasticity above one (Kleiman, 1974; Newhouse, 1977; Parkin et al., 1987; Gbesemete and Gerdtham, 1992; Gerdtham et al., 1992a,b). This strand of literature emphasized on measuring the size of income elasticity of health care (HC), and the policy effects for the investing and delivery of HC resources. On one hand, supporters of health spending being a luxury commodity claim that it is a good same as other goods and should be put up for market forces. Whereas, advocates of HCE being a necessity, stress the role of government control and intervention in the delivery of health care (Culyer, 1989; Di Matteo, 2003). Many of the past studies either have the issue of variables omission and conversion factor procedures or some methodological problems (Kleiman, 1974; Newhouse, 1977). These studies considered income as a main contributing factor in explaining variations in HCE. However, many researchers in the aftermath incorporated non-income variables as determinants of HCE and investigated the effects of these variables on HCE. Age structure of population was identified as a key indicator to explain changes in HCE across nations (Leu, 1987; Cuyler, 1988). The share of population lees than 15 years of age and elderly population such as 65 years and over or up to 75 years of age was estimated in the model while explaining changes in the HCE per capita. It was noted that, these variables showed marginal influence on HCE (Grossman, 1972; Leu, 1986; Di Matteo and Di Matteo, 1998).

#### Technology' role in health care expenditure

Technological progress is one of the key factors to explain variations of HCE (Newhouse, 1992). However, little attention has been paid to the research in order to study the effects of technological progress on HCE, due to the non-availability of an appropriate proxy to capture variations in the medical care technologies. Various proxies have been used in the past studies, such as surgical methods and specific equipment (Baker and Wheeler, 1998; Weil, 2007), health care specific research and development expenditure (Okunad and Murthy, 2002); infant mortality and life expectancy at birth (Dreger, 2005); time index as a proxy for the impact of technology change (Gerdtham and Löthgren, 2000); time-specific intercepts (Di Matteo, 2004). Innovations in technology along with weak cost containment policy were identified as a main contributing factor in increasing health care cost. Major improvements in the health-related technologies, in general, increase health care (Bodenheimer, 2005). The addition of new low cost per patient per year technology to the health care system increases spending on health and health care, because more people are being treated (Lubitz, 2005). Advances and diffusions in medical care technology into the health care systems were the major responsible factors for growing expenditure in health care (Newhouse, 1992). However, there was a conflicting and complex relationship between medical technology and HCE (Sorenson et al., 2013). Health care technology increased survival rates on one hand, but on the other hand it has rapidly increased the cost pertaining to health care as a ratio to GDP (Chandra and Skinner, 2012). Properly managed use of technology, especially mobile technology could be a major driver to cut cost, improve safety, and as a source of improved decision making for health care professionals (Junglas et al., 2009). Medium and long term forecast models try to elucidate the responsible and driving factors of heath care spending for more clear policy choices available. Expenditure growth is more related to budget decisions. Varying innovative technologies contributed more to growth in the medium term as was the case with targeted biologicals (Jakovljevic, 2015), diagnostic radiology (Ranković et al., 2013) and radiation therapy of cancer (Jakovljevic et al., 2014). Various risk factors such as obesity, and variations in the chronic diseases prevalence turned out to be important in the long term forecasting (Thorpe et al., 2004).

#### Aging population and health care expenditure

It is generally anticipated that aging population would be a key factor in health care system and HCE in the near future (United States Congressional Budget Office, 2007). It is also likely that future trends in the health care and long run care would be different (Spillman and Lubitz, 2000). The impact of aging population in the health care spending growth has been extensively investigated in the past studies (Zweifel et al., 1999; Hogan et al., 2001). The Non-significant impact of aging population was found on total per capita HCE (Hoover et al., 2002; Tchoe and Nam, 2010). However, Breyer and Felder (2006), Schulz et al. (2004), (Ogura and Jakovljevic (2014) and Khan et al. (2015) identified aging population as a contributing factor of accruing health care costs; a positive relationship between aging and HCE in short run (Bech et al., 2011). As population ages, total health care spending and expenditure on care of elderly growing (Häkkinen et al., 2008; Mao and Xu, 2014). Aging population was found as a major driver for the health care demand, and thus, an increasing source of health care spending (Reinhardt, 2003). Aging population and aggregate HCE were found to be negatively correlated (Palangkaraya and Yong, 2009). In contrast, the micro-simulation method study showed that there is no sizable impact of aging on health care spending, but the upward movements in the variations were because of prevailing practices (Dormont et al., 2006). However, some studies found that the costs associated with age profile have a tendency to increase to reach a certain level but decline after that level (Martins et al., 2006; Przywara, 2010). Other micro-level studies, (Lubitz et al., 1995; Seshamani and Gray, 2004; Breyer and Felder, 2006) focused on testing whether age or time to death (hereafter, TTD) is responsible for explaining increasing trends of variations in health care spending. Several individual-level studies has suggested that acute health care cost increased as TTD reached nearer and nearer, (Zweifel et al., 1999; Hoover et al., 2002; Breyer and Felder, 2006). However, no significant association found between general practitioners and TTD (Madsen et al., 2002).

A little attention has been paid to find the relationship between the costs of age profile of dying and health care expenditure (Lubitz et al., 1995; Kovacević et al., 2015). Seshamani and Gray (2004) investigated the relationship and found that cost of dying due to acute health care declined beyond a particular age. They found that the reduced acute health care expenditure reflected the possibility of aged populace hospitalization and /or minimizing the chances of introducing intensive treatment.

In many earlier studies, population forecasts and various assumptions were the building blocks for forecasting healthcare or long-term health care cost, particularly, with respect to time spent in good health (Spillman and Lubitz, 2000; Madsen et al., 2002; Breyer and Felder, 2006; Martins et al., 2006). The results of these studies based on time series data were dependent upon the variations in demand as well as on supply sides. Therefore, the estimated HCE be interpreted with respect to utilization of health care instead of demand for health care. On the other hand, projection results were based on certain assumptions made to future age-specific health issues, which were supply side changes. In most of the projections, the utilization rate of age-specific and health care allocation was considered to be constant, and so, could be interpreted from demand side projections. A large portion of this ever increasing demand for medical services appears to be driven by non-communicable prosperity diseases even in top performing BRICS markets (Jakovljevic, 2016).

## Data, model specification, and methods

### Data sources, variables, and their description

This study used annual time series data ranging from 1981 to 2014. Data on relevant variables was collected from various data sources such as Statistics Division Malaysia, World Development Indicators (World Bank Group, 2014), Statistical, Economic and Social Research and Training Centre for Islamic Countries (SESRIC), and Asian Development Bank (ADB) and other published reports. The variables and their description are given in Table 1.

**Table 1:** **Variable names and descriptions**.

**Variables**	**Description**	**Sources**
gdp, gdp_t−1_	Gross domestic product/capita and its lag value in real term	WDI^a^/WHO^b^/ADB^c^
he, he_t−1_	Healthcare expenditure /capita and its lagged values in real term	WDI/SESRIC^d^/ADB
pop_65+_	Share of population age 65 years and above	WDI/SESRIC/ADB
pop_15_	Share of the population under 15 years	WDI/SESRIC/ADB
Le	Life expectancy	WDI/SESRIC/ADB
Popgw	Population growth rate	WDI/SESRIC/ADB

a *WDI, World Development Indicators (World Development Indicators (WDI), December 2015)*.

b *WHO, World Health Organization (http://www.who.int/GHO/)*.

c *ADB, Asian Development Bank (http://www.adb.org/data/statistics)*.

d *SESRIC, Statistical, Economic and Social Research and Training Centre for Islamic Countries (http://www.sesric.org)*.

This study included aging population, as a key driver of HCE because when population ages, public spending as a percentage of GDP is likely to increase, and increased life expectancy is expected to be linked to a decreasing population health status and output (Cuyler, 1988). Life expectancy^1^, real per capita HCEs, real GDP per capita, GDP growth rate^2^, and share of population under age 15 years and 65 years^3^ and above are other variables used in the study. The brief description and data sources are given in the following table.

### Model specification for health care expenditures

The general HCE model can be specified as:

(1) $$\begin{array}{l} hce_{t}=\beta_{0}+\beta_{1}hce_{t-1}+\beta_{2}gdp_{t}+\beta_{3}gdp_{t-1}+\beta_{4}le_{t}+\beta_{5}pop_{t65}+\\ \;+\beta_{6}pop_{t15^{-}}+\beta_{7}popgw_{t}+\varepsilon_{1t} \end{array}$$

(2) $$\begin{array}{l} gdp_{t}=\alpha_{0}+\alpha_{1}gdp_{t-1}+\alpha_{2}hce_{t}+\alpha_{3}hce_{t-1}\\ \;+\alpha_{4}le_{t}+\alpha_{5}pop_{56^{+}t}+\varepsilon_{2t} \end{array}$$

^4^

where

*hce*_*t*_, *hce*_*t*−1_ = real per capita health care expenditure^5^ at time *t* and *t*-1

*gdp*_*t*_, *gdp*_*t*−1_ = is GDP per capita in real term at current time and one period lag

*le*_*t*_ = is life expectancy at birth

$pop_{t65}^{+},pop_{t15}^{-}=$ Population age 65 years and above, and age less than 15 years *and*

*popgw*_*t*_ = population growth at time *t*

ε_*it*_ = are independent and identical distributed (*iid*) error terms.

In Equation (1), HCE acts as an endogenous variable (*hec*_*t*_), a function of GDP per capita and other exogenous variables explained as above. In the Equation (2), real GDP per capita (*gdp*_*t*_) acts as endogenous and HCE as exogenous variable with other explanatory variables.

The β_1_ and β_3_ are adjustment parameters that could be converged to the equilibrium level if there is any shock to the system, and its value lies between 0 and 1. According to economic theory as the real per capita income increases, the expenditure on healthcare is expected to rise, hence β_2_>0. Increasing life expectancy indicates increased overall health conditions of the general public of an economy that could be due to the provision of advanced technologies in the healthcare sector, which have a positive influence on health care spending, so β_4_ > 0. The β_5_ and β_6_ represent the cohort of the population under the age group of 65 years and above and ages less than 15 years; and as the shares of these two cohorts increase the expenditure on health and health care is likely to increase due to more demand for health care services. 0〈α_1_〈 1 and 0〈α_3_〈 1 are the adjustment coefficients; α_1_〉0 because increased health spending enhances good health which as a result increases economic growth; α_4_〉0 because increased life expectancy is a measure of technological changes, which enhances labor force skills and efficiency. The more the skilled and technologically well-versed labor forces the more the efficiency which ultimately spurs GDP per capita. The effect of Population structure on GDP per capita somewhat acts differently and may have negative effect on GDP per capita as more the population ages the dependency ratio increases which affects the income per capita at household levels as well as at country aggregate level. However, if the share of aging population is healthy and participate actively in the economy, then economic growth increase.

### Methods

Based on the objectives of the paper, in the first step, we check the variables for the existence of possible unit root problem in order to get rid of spurious results and for appropriate policy relevance. To this end, we applied ADF and PP individual unit root tests. In the second step, we applied ARDL Bound test to find the short and the long-run relationship between the variables. The variables were checked through various diagnostic tests in order to get reliable, unbiased and consistent estimates of the parameters. Thus, at the final stage to estimate long and short run dynamics equilibrium relation among the series, we used the Unrestricted Vector Error Correction Model (UVECM), and the short and the long run causality were investigated by Granger (1969). During Causality test finally, we employed Granger causality test to investigate the causal link between the variables.

## Results and discussion

### Graphical investigations

**Figure 1:**
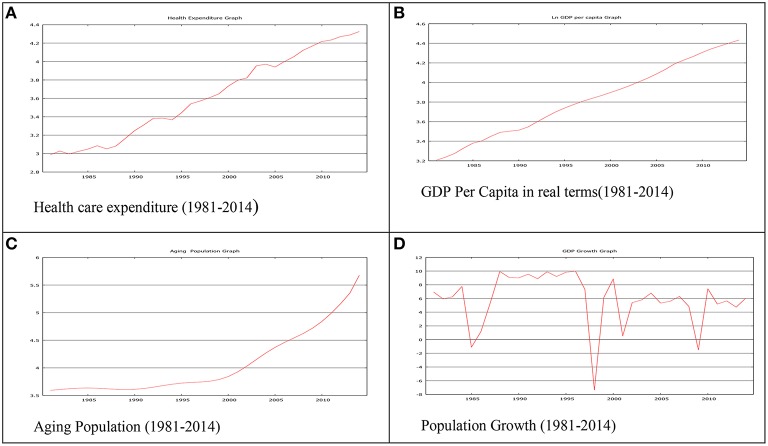
**Graphical investigation. (A)** Health care expenditure (1981-2014). **(B)** GDP Per Capita in real terms(1981-2014). **(C)** Aging Population (1981-2014). **(D)** Population Growth (1981-2014).

Figure 1A showed an upward trend over time with drift. So, the series, HCE a non-stationary series. Figure 1B showed that GDP per capita is upward trending with non-stationarity behavior without drift. Figure 1C of aging population exhibits steady increase from 1981 to 2000, but afterward drifting with a trending pattern, which clearly shows time-variance. Looking at the above Figure 1D of population growth since 1981, no prominent pattern exists in the data, so the data generating process showing random walk with drift and some irregularities during 1985 to 1995 and low growth rate in 1998, 2001 and 2008-2009.

### Unit root test

The ADF and PP unit root test results show that the variables are mixtures of I(0) and I(1) and no variable is found to be I(2), however, the results are not reported here in order to save the space and can be obtained on request.

### ARDL co-integration results

Since the unit root tests show a mixture of I(0) and I(1) variables and none of the variables is I(2), which is the pre-assumption for application an ARDL bound test. The bound test results confirm a long co-integration relationship between HCE and income per capita and the data series pass all the relevant diagnostic tests. These results can be provided on request as has not been shown due to space limitations.

### Regression results

Table 2 summarized the estimated results of the regression taking HCE as a dependent variable, regressed on other explanatory variables as shown in the Equation (1). All the variables significantly affecting health expenditure, however, the impact of GDP growth was non-significant. The estimated signs of the variables were consistent as reported in the past literature. Income per capita has a positive and significant influence on HCE and the estimated elasticity value for HCE was 0.995502 < 1. The value of income elasticity value less than one indicated that HCE was still treated as a necessity good (Khan et al., 2015). The highest t-ratio of income per capita showed that it was a major contributing factor responsible for explaining variations in HCE. Therefore, as the real per capita income of the general public increases, people spend more on their health in order to keep themselves healthier, active, and live longer lives. This result is consistent with previous studies, conducted either at the individual country level or as a panel of countries, for example, (Newhouse, 1977; Parkin et al., 1987; Gbesemete and Gerdtham, 1992; Gerdtham et al., 1998; Dreger, 2005; Khan et al., 2015). The impact of population growth and population structure was also significant. Negative signs of the population with the age group under 15 years and age group of 65 years and above were according to the expectations and are consistent with the earlier research (Khan et al., 2015). The negative influence of the population structure showed increase dependancny ratio on the working age group, which might put pressure on the overall GDP of the country and on allocation of resouces to other sectors of the economy. The possible reason could be that people in this two cohort, spend more on their health either from their past savings or household expenditure. The 15 years and below age group contributes lesser to the development of the economy as they are not actively engaged in economic activities. This age group spends more on health and earns less. Similarly, the elderly population in the declining periods of life are more susceptible to illness, thus, they need health care to keep themselves healthy (Ogura and Jakovljevic, 2014; Khan et al., 2015). This result implies that for people over age 65 proximity of death may increase, which eventually reduces short run HCE in Malaysia. This result is consistent with (Erdil and Yetkiner, 2009; Ogura and Jakovljevic, 2014; Khan et al., 2015). The negative relationship between health care and population growth could be due to the non-random pattern of the population growth. As was obvious from the Figure 1D where the growth rate significantly drops down over certain time periods, which outpaces the positive effects.

**Table 2:** **Model: OLS results [1981-2014 (*T* = 34)] Dependent variable: lnhce**.

**Var**	**Coeff**	***SE***	**T-ratios**	***P*-value**
Constt	0.960535	0.245831	3.9073	0.00054^*^
Lngdpp	0.995502	0.044104	22.5713	< 0.00001^*^
Popgr	−0.31141	0.038114	−8.1702	< 0.00001^*^
Pop15	−0.10281	0.018512	−5.552	0.00264^*^
Pop65	−0.10827	0.028645	−3.7798	0.00076^*^
Gdpgr	0.000317	0.001738	0.1826	0.85640
Le	0.567102	0.073540	7.71148	0.00016^*^

*R-squared: 0.994967 and Adjusted R-squared: 0.994068*.

* *Shows significant at 5 percent and 1 percent significant level*.

## Conclusion

The rapid increasing pattern of HCE in Malaysia is a serious concern for the policy makers as well as the decision makers. The purpose of this paper is to model the determinants of HCE and find the effects of these determinants on HCE in the time series framewok from 1981 to 2014 in Malaysia. For the empirical investigation, the paper used ADF, PP tests to check for unit root issue and the ARDL approach for co-integration. For causality between HCE and GDP per capita Ganger VECM is applied. For long run parameters estimation an Ordinary Least Square (OLS) regression is used. The unit root tests confirm mixture of integrating order of the variables, i.e., I(0) and I(1). The ARDL Bound test shows the presence of a long run co-integration between HCE and GDP per capita. The rgeression results confirm that income per capita has a positive and significant effect on HCE with income elasticity for HCE 0.999 < 1. The income elasticty value shows that HCE is a necessity. The closest value of income elasticity for HCE to one is the most important and intersting result of the study in the case of Malaysia for the sampled period. This indicates that Malaysia is the most rapidly growing economy in the Association of southest nations (ASEAN) and in the near future would be in the list of developed countries. Moreover, population growth and population structure have significant negative impact on HCE. The effect of technological changes, proxy by life expectancy also has a positive and significant influence on HCE. Thus, GDP per capita in real term, population growth, population strucutre and technology is the major contribtor to explain variations in HCE in Malaysia. The Ganger VECM results also comfirm a bidirectional causality between HCE and real GDP per capita.

The findings of the paper provide an insight to the policy makers that health expenditure play a significant role in the economic development of Malaysia. Therefore, to create healthy, efficient, technologically skilled and productive labor force it is suggested that encouraging HCE policies be adopted in Malaysia. The Ministry of Health Malaysia (MOH) should provide basis health facilities as well as promote health education to the common people of the country with a special emphasis on rural health.

## Author contributions

All authors listed, have made substantial, direct and intellectual contribution to the work, and approved it for publication.

### Conflict of interest statement

The authors declare that the research was conducted in the absence of any commercial or financial relationships that could be construed as a potential conflict of interest.

## Footnotes

^1^Life expectancy is used as a proxy to capture the effect of technology change on health care expenditure. A change in the levels of life expectancy has influence human behavior at individual and aggregate level. It also impacts fertility, human capital, growth process (Coile et al., 2002).

^2^Lagged values of the health care and gdp are included as previous year values effect current expenditures.

^3^Share of population under age 15 and 65^+^ years is used to investigate the influence of changing population.

^4^Gross domestic product (GDP) is modelled in Equation (2) to find the causal effect if any between health care expenditure and GDP.

^5^All the variables are transformed into natural log and model the data in the linear log form for interpreting the estimated results in elasticities and for allowing diminishing marginal returns to other explanatory variables.
